# Anti-Wetting PVDF Membrane Modification by Coating Fluoride and Deposing Different Silicon Contents for Membrane Distillation Treatment of Ammonia Nitrogen Wastewater

**DOI:** 10.3390/membranes16030100

**Published:** 2026-03-06

**Authors:** Qianliang Liu, Xin Guo, Hengyu Ai, Hongbo Liang, Fen Li, Caihong Liu

**Affiliations:** 1 Key Laboratory of Green Chemical Engineering and Technology of Heilongjiang Province, School of Material Science and Chemical Engineering, Harbin University of Science and Technology, Harbin 150080, China; liuqianliang@hrbust.edu.cn (Q.L.); 15945362821@163.com (X.G.); lifen@hrbust.edu.cn (F.L.); 2 School of Mining Engineering, Heilongjiang University of Science and Technology, Harbin 150022, China; lhbhljkjdx@163.com; 3 Key Laboratory of Eco-Environments in Three Gorges Reservoir Region, Ministry of Education, College of Environment and Ecology, Chongqing University, Chongqing 400044, China

**Keywords:** membrane distillation, anti-wetting modification, source-separated urine, landfill leachate, ammonia nitrogen recovery

## Abstract

Membrane distillation (MD) was a promising approach for treating highly concentrated ammonia–nitrogen wastewater. However, membrane wetting often limited large-scale application. To address this, we built an anti-wetting layer on a commercial PVDF membrane surface by coating fluoride and depositing SiO_2_ nanoparticles. Three PVDF/ SiO_2_/F membranes were prepared with different silicon contents: 1%, 6%, and 12% (volume) of tetraethyl orthosilicate (TEOS). These processes created different surface roughness on the modified membranes. Results showed that the membrane containing 6% TEOS exhibited the best resistance to sodium dodecyl sulfate (SDS) in NaCl solution. This optimized membrane was subsequently tested with real wastewater, including source-separated urine and landfill leachate. In 10 h, it removed 97.5% of total organic carbon (TOC) from urine, achieving an ammonia absorption rate of 55.1% and removed 92.4% from leachate, with an ammonia absorption rate of 37.58%. These results provide a reference for membrane fabrication parameter optimization to enhance the membrane’s anti-wetting ability.

## 1. Introduction

Highly concentrated ammonia wastewater represents both an environmental pollution and a potential nutrient resource. Developing effective strategies to treat and recycle high-ammonia nitrogen wastewater became a key research focus. This approach not only mitigated environmental pollution but also facilitated resource recycling, such as converting nitrogen into fertilizers [1,2], thereby generating significant environmental and economic benefits. This kind of challenging wastewater originated from multiple sources. Typical examples included source-separated urine [3] and landfill leachate [4,5,6], which had high ammonia nitrogen content. Treatments for ammonia removal generally adopted biological processes, such as MBR [7,8,9] or anammox [10,11,12], and physicochemical processes, including struvite precipitation [13,14,15] or air stripping [16]. In addition, membrane technologies could effectively remove most organic pollutants and total nitrogen (TN). For example, nanofiltration (NF) [17,18,19], ultrafiltration (UF) [20,21], and reverse osmosis (RO) [22,23,24,25,26] were the primary membrane technologies employed in the treatment of landfill leachate.

Membrane distillation, as an emerging membrane separation technology, demonstrated significant advantages for its unique thermally driven mechanism and non-extra pressure or low-pressure conditions. Compared to traditional pressure-driven membrane processes, MD not only efficiently concentrated high-salinity wastewater but also exhibited low membrane fouling tendency. These advantages have given MD wide prospects for application in treating actual wastewater [27,28,29,30,31,32,33,34], yielding pure water [35,36] and desalination [37,38,39,40,41,42], especially ammonia nitrogen separation and recovery [43,44]. In high concentrated ammonia–nitrogen wastewater treatment, MD exploited the high volatility of ammonia in high pH condition. The volatile ammonia vapor passed through the membrane’s hydrophobic pores and was captured on the permeate side by an absorption liquid, typically dilute sulfuric acid, while non-volatile contaminants, such as salts and most organic compounds, remained in the feed solution. This mechanism achieved efficient ammonia removal and recovery under mild operating conditions and overcame limitations commonly encountered in high-pressure systems, including low ammonia separation efficiency, concentration polarization and intensified membrane fouling. In this process, several operating parameters influenced ammonia transfer and absorption efficiency, such as the pH, the temperature of feed and permeate, feed concentration, and absorbent concentration. Despite these potentials, MD still faced material-related challenges when applied in actual wastewater treatment. Conventional hydrophobic membranes such as PVDF and PTFE, which were the most commonly used in MD, were prone to wetting and fouling by organic contaminates during operation. Progressive wetting and fouling could reduce membrane performance and drastically impair contaminant rejection and water vapor transport. Consequently, efforts had focused on enhancing membrane resistance to fouling and wetting [45,46,47,48,49,50].

For thermally driven MD processes, enhancing the hydrophobicity of membrane surface was one of the main methods to improve their anti-fouling and anti-wetting properties. Surface fluorination [51,52] is commonly used to reduce membrane surface energy, thereby increasing hydrophobicity and chemical stability. Fluorinated functional groups effectively suppress liquid intrusion into membrane pores, which is particularly advantageous under complex feed conditions such as high-salinity [53] and highly concentrated ammonia wastewater [54]. Meanwhile, incorporating nanoparticles [55,56], such as silica, creates micro- and nano-scale surface roughness, enhancing the hierarchical structure and promoting air entrapment at the membrane–liquid interface. Together, fluorination and silicon-induced roughening provide a synergistic effect, stabilizing the Cassie–Baxter wetting state and effectively suppressing pore wetting under harsh MD operating conditions. For example, Lu et al. [57] coated a super-thin Teflon layer (0.025 wt% Teflon^®^ AF2400, Sigma–Aldrich, Darmstadt, Germany) on PVDF hollow fiber membranes. This modification increased the contact angle from 105° to 151°, confirming the successful formation of superhydrophobic membrane surface. In desalination tests, the modified membrane achieved 99.99% salt rejection and maintained a stable flux of 21.1 kg/(m^2^·h) for over 250 h. Li et al. [58] spray-coated PVDF membranes with a mix of fluorocarbon surfactants, silanes, and silica nanoparticles. The resulting composite membrane exhibited increased surface roughness, reduced surface energy, and complete hydrophobicity. It also demonstrated nearly complete rejection (about 99.99%) and excellent resistance to both wetting and fouling. Similarly, Xiong et al. [59] modified PTFE membranes by attaching silica nanoparticles followed by fluorinated coating. The modified superhydrophobic membrane exhibited extremely low surface energy (1 mJ/m^2^) and outstanding anti-wetting properties. Fan et al. [60] adopted a different strategy by fabricating a dual-layer membrane through phase separation and sol-gel methods, incorporating silica nanoparticles and fluorochemicals. This approach produced an ‘amphiphobic’ surface, which was capable of repelling both water and oil phases. Even in conditions of containing surfactants such as SDS, this membrane maintained stable distillation performance and outperformed commercial hydrophobic membranes.

Although surface modification was widely used to enhance membrane roughness, the influence of silicon content, especially SiO_2_ nanoparticle loading, on membrane surface properties and MD performance had not yet been fully researched. Moreover, the application of modified membranes for ammonia–nitrogen recovery from actual highly concentrated ammonia wastewater had rarely been applied. In this work, we developed a modified membrane with optimal silicon content and evaluated its performance using real wastewater containing high ammonia concentrations. A commercial PVDF membrane served as the substrate for grafting SiO_2_ nanoparticles, followed by a fluoride coating to stabilize surface hydrophobicity and enhance anti-wetting performance during MD treatment of highly concentrated ammonia wastewater. The optimization of parameters related to varying SiO_2_ loading capacities during the roughness preparation process was proposed. The MD performance of modified membranes was investigated for surfactant-containing saline water and simulated high concentration ammonia–nitrogen wastewater. Moreover, we applied the optimal modified membrane to source separated urine and landfill leachate (represented actual high concentrated ammonia wastewater). The membrane’s performance of anti-wetting and ammonia recovery were evaluated for the feasibility of long-term stable operation.

## 2. Materials and Methods

### 2.1. Membrane Preparation

**Figure 1:**
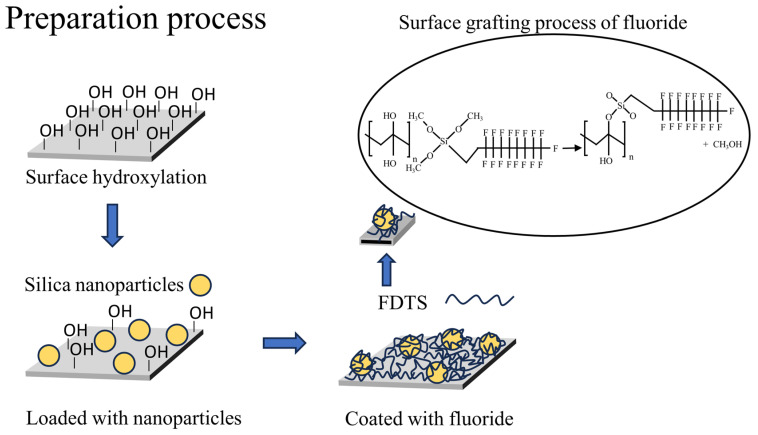
Preparation process for superhydrophobic PVDF/SiO_2_/F composite membranes (corresponding to M1, M2, and M3) and the surface grafting process of fluoride.

In this experiment, PVDF/F and PVDF/SiO_2_/F membranes were fabricated. Fluorination was employed to maintain surface hydrophobicity, while the incorporation of silica was aimed at enhancing surface roughness. The preparation process of the PVDF/SiO_2_/F composite membrane was shown in Figure 1. A commercial PVDF sheet membrane (0.45 μm pore size, Millipore) was used as the substrate. The dried PVDF substrate membranes were immersed in a 7.5 mol/L NaOH solution at 60 °C for 3 h to hydroxylate the surface (Figure 1). After hydroxylation treatment, membranes were rinsed thoroughly with deionized water to remove residual alkali, then dried in a 40 °C oven for 1 h before use. SiO_2_ gels prepared from three different initial TEOS contents (1%, 6% and 12%) were coated onto the surface of the hydroxylated membrane. After complete drying, the fluorinated solution was applied to graft the fluorinated groups onto the composite membrane surface. The coated membranes were subsequently dried in a 40 °C forced-air oven for 6 h, producing three PVDF/SiO_2_/F composite membranes with different surface silicon contents. For comparison, PVDF/F composite membranes were fabricated under identical conditions, but without SiO_2_ gel loading.

To simplify the presentation of experimental results, the nomenclature listed in Table 1 was used throughout the subsequent discussion. The procedures for preparing the SiO_2_ gel and fluoride solution are detailed in the Supplementary Materials (Text S1).

### 2.2. Membrane Characterization

The surface morphology and microstructure of the hydrophobic membranes were characterized using a cold field-emission scanning electron microscope (SEM; Hitachi Regulus 8100 series, Hitachi, Tokyo, Japan). Surface roughness was analyzed by an atomic force microscope (AFM; Dimension Edge series, Bruker, Billerica, MA, USA), and the average roughness (R_a_) and root mean square roughness (R_q_) were calculated according to Equations (1) and (2) in Supplementary Materials (Text S2).

The chemical elements and contents on the hydrophobic membranes surface were determined by an X-ray photoelectron spectrometer (XPS; Escalab 250Xi, Thermo Scientific, Waltham, MA, USA). The static water contact angle of the membranes was measured by a contact angle goniometer (OCA 20, Dataphysics, Filderstadt, Germany). The specific surface area and pore structure of the membranes were analyzed using a fully automatic surface area and porosity analyzer (BET; ASAP 2460 series, Micromeritics, Norcross, GA, USA).

### 2.3. DCMD Experiments

#### 2.3.1. DCMD Performance

**Figure 2:**
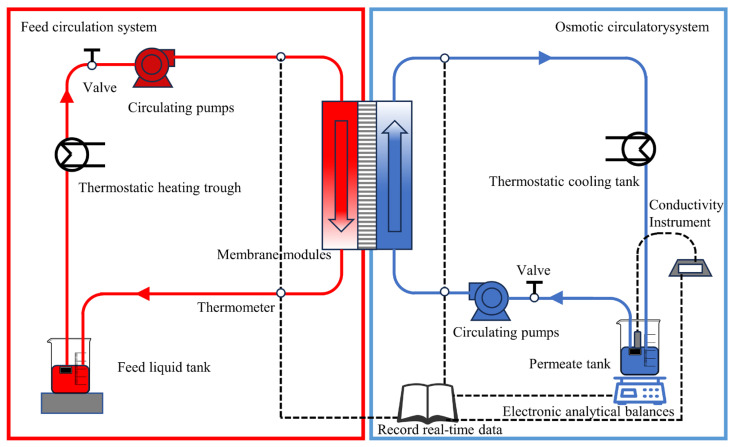
Diagram of DCMD system.

Direct contact membrane distillation (DCMD) experiments were conducted using a custom-built apparatus comprising a rectangular membrane module, feed and permeate circulation loops, two temperature control systems, and a weight monitoring unit (Figure 2). The membrane measured 2.5 cm in length and 1.5 cm in width, with an effective area of approximately 3.75 cm^2^. The DCMD experimental details are provided in the Supplementary Materials (Text S3).

To evaluate the MD performance of the modified PVDF/SiO_2_/F composite membrane, a 3.5 wt% NaCl solution was used in the feed side, while deionized water was used in the permeate side. The temperature difference across the membrane was maintained at 50 °C (70 °C on the feed side and 20 °C on the permeate side), with a cross-flow velocity of 0.01 m/s. Each test was run for 10 h. Membrane distillation performance and wetting resistance were assessed by monitoring membrane flux and conductivity of the permeate.

The flux of membrane distillation was calculated by Equation (1)

(1) $$J=\frac{W_{t}}{A_{m}\cdot t}$$

where W_t_ is the mass difference of the permeate in t time (kg); A_m_ is the effective membrane area in the experimental setup (m^2^), and t is the operating time for collecting the condensate (h).

To further assess the anti-wettability of the composite membrane, 0.25 wt% SDS was added to the 3.5 wt% NaCl feed solution to lower surface tension and accelerate wetting. The time at which abnormal changes (weight or conductivity) occurred was recorded during the MD operation.

#### 2.3.2. Ammonia Absorption Efficiency

To further evaluate the ammonia–nitrogen absorption performance of pristine PVDF and modified superhydrophobic membranes, experiments were conducted using simulated ammonia–nitrogen wastewater prepared by ammonium chloride (NH_4_Cl) as feed. Increasing the initial pH of the wastewater could convert the majority of ammonium ions (NH_4_^+^) into volatile ammonia (NH_3_). The resulting NH_3_ concentration gradient across the membrane drove ammonia to permeate through the pores, where it was absorbed by the dilute acid in the permeate side, achieving separation from the feed solution. The operating temperature on both sides of the membrane was the same as 2.3.1. The ammonia–nitrogen absorption efficiency was evaluated under different initial pH values (10–12), feed ammonia–nitrogen concentrations (550–1000 mg/L), and acid concentrations in the absorption solution (0.204–0.51 mol/L). The ammonia–nitrogen absorption rate was calculated according to Equation (2).

(2) $$\alpha_{N}=(1-\frac{C_{p,t}\;\times \;V_{p,t}}{C_{f,0\;}\times {\;V}_{f,0}})\;\times \;100\%$$

where α_N_ is the ammonia nitrogen absorption rate %; C_f,0_ is the initial ammonia nitrogen concentration on the feed side (mg/L); V_f,0_ is the initial feed side volume (L); C_p,t_ is the ammonia nitrogen concentration on the permeate side at t time (mg/L); and V_p,t_ is the permeate side volume at t time (L).

Additional water quality parameters monitored during the experiments included total organic carbon (TOC), pH, and conductivity. TOC was measured using a total organic carbon analyzer (TOC-L, Shimadzu, Beijing, China), while pH and conductivity were determined using a pH meter (pHS-3C, Leici, Shanghai, China) and a conductivity meter (DDS-307F, Leici, Shanghai, China), respectively.

#### 2.3.3. DCMD Experiments for Real Wastewaters

To assess the anti-wettability and surface stability properties of modified membranes for real wastewater treatment, real urine and landfill leachate (containing high concentrations of organic matter and ammonia–nitrogen) were used in MD experiments. The ammonia–nitrogen absorption efficiency and stability of M0 and M2 membranes were evaluated. During these MD experiments, the permeate weight, ammonia–nitrogen, and TOC concentrations were measured. The TOC retention rate for MD process was calculated by Equation (3).

(3) $$R_{\mathrm{TOC}}=(1-\frac{C_{p,\mathrm{TOC}}}{C_{f,\mathrm{TOC}}})\;\times \;100\%$$

where: R_TOC_ is the TOC retention rate, %; C_f,TOC_ is the TOC concentration on the feed side, (mg/L); C_p,TOC_ is the TOC concentration on the permeate side, (mg/L).

## 3. Results and Discussions

### 3.1. Membrane Surface Analysis

#### 3.1.1. Membrane Surface Characterization

**Figure 3:**
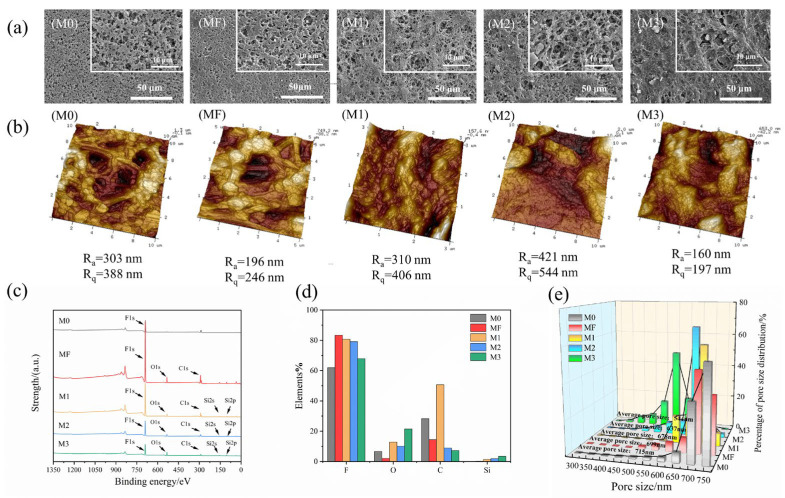
(**a**) SEM images magnified 2000 times and 10,000 times; (**b**) AFM images; (**c**) Percentage distribution of pore sizes; (**d**) XPS spectra; (**e**) Elemental percentages.

The surface morphology of the M0, MF, M1, M2, and M3 membranes was examined by SEM, and the results are shown in Figure 3. As shown in Figure 3a, the pristine PVDF membrane (M0) displayed a dense and uniform fibrous network formed by interwoven fibers. After surface hydroxylation and fluoropolymer grafting, the surface structure became partially blocked and covered by fluoropolymers. However, most original pores remained intact, allowing the transmembrane vapor transport.

The silicon–fluorine composite membranes (M1, M2 and M3) were shown in Figure 3a (M1, M2, M3). After modification, the originally uniform and dense surface structure had significant changes. With increasing loading of SiO_2_ nanoparticles, pore blockage on membrane surface became more obvious. However, compared with M1 and M3, the nanoparticles in the M2 presented a relatively even distribution leading to a uniform and dense covering layer. For M1, due to the low SiO_2_ loading, the particles were sparsely attached on the PVDF surface and partially covered the pores. Also, due to the overloading of SiO_2_, the original surface of M3 was almost completely covered, which might significantly increase the membrane’s mass transfer resistance.

R_a_ and R_q_ of the M0, MF, M1, M2, and M3 membranes were illustrated in Figure 3b. After fluorination modification, the average roughness of the membrane surface decreased, likely due to the adhesion of fluorinated compounds that smoothed the membrane surface. For the membranes loading SiO_2_ nanoparticles, the M2 membrane had an R_a_ of 421 nm and an R_q_ of 544 nm, representing a substantial roughness increase compared with M1 and M3. This result provided further evidence that more nanoparticles were loaded onto the M2 surface than M1. However, the M3 membrane had an R_a_ of 160 nm and an R_q_ of 197 nm, respectively, which were lower than both the pristine PVDF and other composite membranes, indicating a reduced average roughness. This reduction likely resulted from severe nanoparticle agglomeration on the M3 surface, which blocked most pores and flattened the previously uneven texture.

XPS was employed to analyze the surface chemical composition and elemental content of the M0 and MF membranes, confirming the successful grafting of fluorine groups on the membrane surface (Figure 3c). For M0, the characteristic peaks at binding energies of 685 eV, 532 eV, and 285 eV correspond to F 1s, O 1s, and C 1s, respectively. After surface fluorination, the intensity of the F 1s peak was increased markedly, and increases in binding energies of –C–O and –CF_3_ groups were observed. Meanwhile, the –CF peak shifted from 289.4 eV to 290.6 eV, indicating the formation of new –CF_2_ groups. For the M1, M2, and M3, new peaks at 152 eV and 103 eV were observed, corresponding to Si 2s and Si 2p, indicating the successful incorporation of SiO_2_ nanoparticles onto the membrane surface (corresponding to Figure 3a, M1, M2, M3). The elemental composition derived from the XPS spectra was shown in Figure 3d. The content of F and Si in the composite membranes increased, confirming the successful grafting of fluorine and SiO_2_ nanoparticles onto the PVDF membrane, with the gradual rise of silicon content from M1 to M3.

BET analysis showed that the average pore size decreased with the increasing amount of SiO_2_ nanoparticles in Figure 3e. The average pore size of M0 was 715 nm, while that of MF was 699 nm. The average pore sizes of M1, M2, and M3 were 678 nm, 637 nm, and 544 nm, respectively, demonstrating a gradual decrease relative to the pristine membrane (M0). The SEM and pore size distribution curves corroborated that higher nanoparticle loading resulted in smaller membrane pores.

#### 3.1.2. Hydrophobic Properties and Stability of Membranes

**Figure 4:**
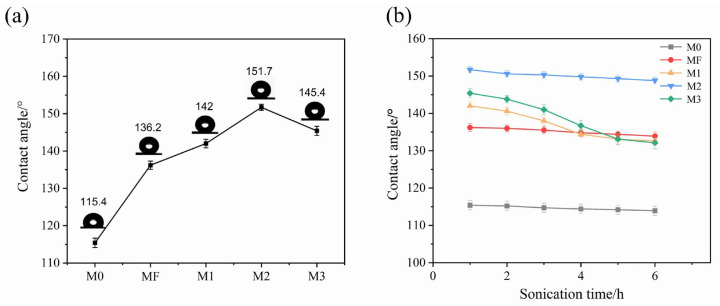
(**a**) Comparison of static water contact angles for membrane surfaces; (**b**) Changes in the contact angles of membranes during ultrasonic treatment.

The hydrophobicity of the membrane surface plays a crucial role in preventing pore wetting. To evaluate hydrophobic performance, the static water contact angles of the pristine and composite membranes were measured and presented in Figure 4a. The contact angle of the M0 was 115.4°, whereas that of the PVDF/F composite membrane increased to 136.2°, indicating that grafting fluorinated compounds significantly enhanced the contact angle, despite a slight smoothing of the membrane surface. After incorporating SiO_2_ nanoparticles and performing surface fluorination, the contact angles of M1, M2, and M3 membranes were 142°, 151.7°, and 145.4°, respectively. Among these, M2 exhibited a contact angle above 150°, confirming the successful formation of a superhydrophobic surface. These results demonstrated that sequential SiO_2_ nanoparticles incorporation followed by surface fluorination could further enhance the membrane’s hydrophobicity.

The improvement was attributed to increased surface roughness caused by SiO_2_ nanoparticle incorporation. According to the Cassie–Baxter model, enhanced surface roughness further increased the static water contact angle of a hydrophobic surface. In addition, the introduction of fluorides formed a thin fluorinated layer that lowered surface energy, further increasing the contact angle. Notably, the M3 membrane showed a reduction in hydrophobicity, likely due to excessive agglomeration of excessive SiO_2_ nanoparticles. Instead of forming a uniform rough structure, these particles aggregated into large clusters, as shown in Figure 3a (M3). Such agglomeration reduced both effective surface roughness and porosity. For the hydrophobic membrane, contact angle and pore size were two key parameters affecting wetting resistance. Generally, a higher contact angle and a smaller pore size were considered favorable for improved anti-wetting performance, as widely reported in the literature [61,62]. In the present study, however, these two parameters did not follow the same trend with increasing SiO_2_ content. Specifically, when the SiO_2_ content increased to 12%, the water contact angle began to decrease, whereas the average pore size continued to decrease monotonically. This indicated that wetting resistance was not governed by a single surface parameter, but rather resulted from the combined effects of multiple membrane characteristics.

The stability of the modified PVDF/F and series of PVDF/SiO_2_/F composite membranes were evaluated by immersing the samples in distilled water under continuous ultrasonic treatment for 6 h. The static water contact angles were measured hourly to monitor the stability of the surface modification layer (Figure 4b). After 6 h, the contact angle of M0 and MF slightly decreased by only 1.3% and 2.3% respectively. For the M1 and M3 membranes, the contact angles slightly decreased during the first hour, followed by an accelerated decline in subsequent hours. After 6 h, M3 exhibited the greatest decrease in contact angle (9.2%), followed by M1 (6.6%). This could be attributed to the high SiO_2_ nanoparticle loading on the M3 membrane, which weakened the surface stability and made the particles prone to detachment. In contrast, the M1 contained more sparsely and unevenly distributed SiO_2_ nanoparticles, making them more susceptible to detachment. Loss of surface particles led to a corresponding decrease in the contact angle. However, due to the lower nanoparticle loading on M1 comparing to M3, the decrease in contact angle was less pronounced.

For the M2, the contact angle decreased by only 2.2% after 6 h of ultrasonic treatment. This stability was attributed to the uniform nanoparticle distribution and their tight encapsulation by fluorides, which minimized coating detachment and enabled long-term membrane stability during operation. Despite the contact angle decrease of M1 and M3, both of them still maintained higher hydrophobicity than the pristine membrane (M0) after 6 h of ultrasonic treatment.

### 3.2. MD Performance Testing

#### 3.2.1. Anti-Wetting Performance of the Membranes

**Figure 5:**
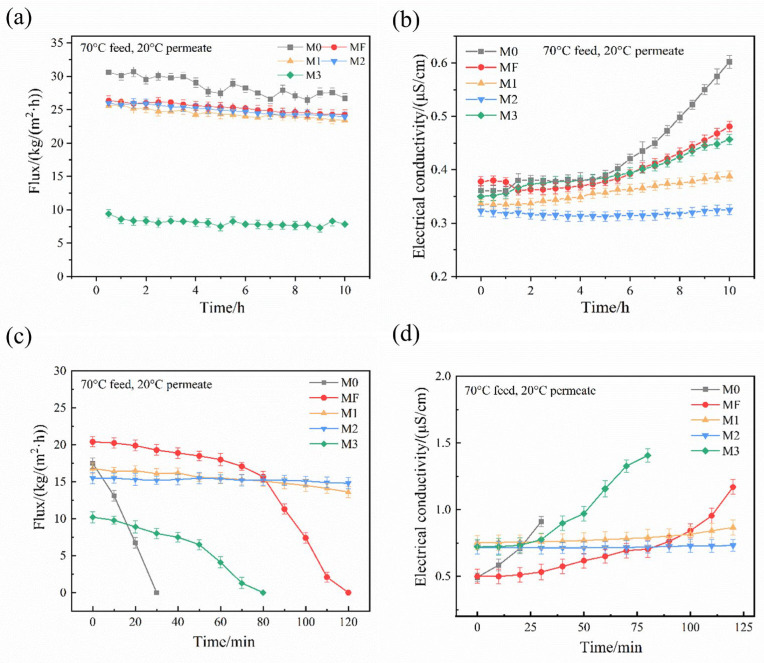
Membrane distillation permeate flux (**a**) and permeate side conductivity (**b**) of the membranes for a 3.5 wt% NaCl in feed solution; Membrane permeation flux (**c**) and permeate side conductivity (**d**) of the membranes for a 0.25 wt% SDS + 3.5 wt% NaCl feed solution.

DCMD experiments were performed on the M0, MF, M1, M2, and M3 membranes to evaluate their desalination and anti-wetting performance after surface modification under the conditions described in Section 2.3.1. The conductivity in permeate side (deionized water) ranged from 0.2 to 1 μS/cm. The variations in membrane flux and permeate-side conductivity over time were shown in Figure 5a,b.

In the case of desalination for 3.5 wt% NaCl feed solution, the average fluxes of M0, MF, M1, M2, and M3 were 28.47, 25.29, 24.41, 24.91, and 8.06 kg/(m^2^·h) during 10h of operation, respectively. It was indicated that coating an extra layer on the membrane surface increased the mass transfer resistance of the membrane, especially for M3 (higher amount of SiO_2_ nanoparticles than other modified membranes). For M0, the flux decreased from an initial 30.6 kg/(m^2^·h) to 26.71 kg/(m^2^·h) over 10 h, corresponding to a 12.7% decline. During the first 5 h, the permeate side conductivity remained stable, but increased sharply from 0.381 μS/cm to 0.602 μS/cm in the following 5 h. MF exhibited a smaller flux decline (7.8%) and stable permeate conductivity, owing to the fluorinated layer’s role in enhancing hydrophobicity and minimizing pore wetting. All three modified membranes (M1, M2, M3) showed lower fluxes than M0, and exhibited a gradual decline in permeate flux during the whole 10 h MD operation. At the end of the test, the fluxes of M1, M2 and M3 decreased by 8.5%, 7.9%, and 16.5%, respectively. The notably lower flux of M3 resulted from partial pore blockage by excessive nanoparticle loading on the membrane surface, consistent with the characterization results in Figure 3a (M3). Meanwhile, the permeate-side conductivity of M1 M2 and M3 all gradually increased (Figure 5b). For M1, conductivity increased from 0.336 μS/cm to 0.388 μS/cm. The smaller increase compared with MF suggested that SiO_2_ nanoparticles enhanced surface roughness and hydrophobicity, thereby improving salt rejection. The permeate-side conductivity of M2 remained stable, with a slight initial decrease, indicating superior separation performance than M1 and M3. These results demonstrated that the M2 exhibited superior anti-wetting properties and higher salt rejection, ensuring stable long-term performance. For M3, the conductivity increase was more pronounced, from 0.35 μS/cm to 0.457 μS/cm, likely due to uneven distribution of surface SiO_2_ nanoparticles, which induced partial pore wetting and facilitated NaCl diffusion into the permeate. Nevertheless, the conductivity increase rate remained lower than that of the pristine PVDF membrane. This indicated that, despite the excessive SiO_2_ loading, the increased surface roughness and enhanced hydrophobicity effectively preserved salt rejection.

To evaluate the anti-wetting performance of the modified composite membranes during DCMD operation, 0.25 wt% of surfactant SDS was added to the 3.5 wt% NaCl feed solution. This addition reduced the solution’s surface tension, enabling further evaluation of the membranes’ anti-wettability and anti-fouling properties. As shown in Figure 5c,d, the permeate flux of M0 rapidly dropped to zero within 30 min, accompanied by a sharp increase in permeate conductivity. This combination indicated that complete membrane wetting occurred, thereby leading to a loss of rejection capability. This was attributed to SDS adsorption on the membrane surface and infiltration into the pores, which hindered water vapor transfer and reduced mass transfer.

Under identical conditions, MF flux decreased by about 23% (from 20.41 kg/(m^2^·h) to 15.7 kg/(m^2^·h)) during the first 80 min. This suggested that the fluorinated surface layer partially prevented surfactant penetration. However, its conductivity subsequently increased sharply after 80 min, and its flux dropped to zero over the next 40 min. Similarly, M3 flux began to decline significantly after 40 min, accompanied by a rapid increase in conductivity, until extensive wetting and a complete loss of mass transfer occurred at 80 min.

In contrast, M1 and M2 maintained stable permeate flux and conductivity throughout the 120 min test. This stability was attributed to their enhanced hydrophobicity caused by moderate SiO_2_ nanoparticle loading, which prevented significant surfactant adhesion. Moreover, the flux reduction in M2 was less pronounced than in M1, and its permeate conductivity remained lower throughout the test. This suggested that M2, as a superhydrophobic membrane with optimal SiO_2_ nanoparticle loading, had excellent resistance to surfactants in the MD process.

#### 3.2.2. Ammonia Recovery Efficiency

**Figure 6:**
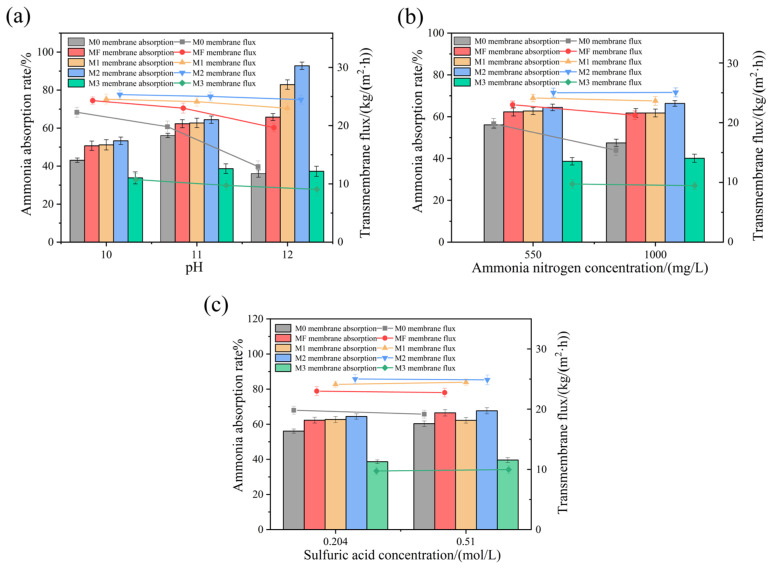
Ammonia–nitrogen absorption rate and permeation flux of the membranes at (**a**) different initial pH values (NH_3_-N: 550 mg/L, H_2_SO_4_: 0.204 mol/L); (**b**) different ammonia–nitrogen concentrations in feed (initial pH: 11, H_2_SO_4_: 0.204 mol/L); (**c**) different acid concentrations in permeate (initial pH: 11 NH_3_-N: 550 mg/L).

The mass transfer process of ammonia–nitrogen was mainly affected both by the ammonia vapor pressure in the feed and the mass transfer resistance of the membrane. Increasing the pH can significantly enhance the vapor pressure of ammonia–nitrogen on the membrane surface. However, a higher pH also promoted hydroxylation on the membrane surface, making it more prone to wetting. Therefore, enhancing the membrane’s resistance to wetting allowed for ammonia removal at higher pH values, thus improving the ammonia–nitrogen mass transfer efficiency. As shown in Figure 6a, the increased pH levels raised the vapor pressure of ammonia, thereby leading to higher absorption rates—a trend observed for the MF, M1, and M2 membranes. However, the M0 membrane exhibited an unexpected decrease in absorption rate at pH 12. This decline was attributed to the high NaOH concentration (used to adjust pH value), which had a hydroxylation effect on the membrane surface, thereby reducing the membrane’s surface hydrophobicity and causing premature wetting. Notably, the M2 membrane demonstrated the most significant improvement, with its absorption rate increasing with pH from 53.3% to 92.8%. The M3 membrane exhibited a much lower absorption rate than M1 and M2 because of its highest transfer resistance, caused by excessive SiO_2_ nanoparticle coating. The increasing mass transfer resistance was the main reason limiting ammonia nitrogen transfer. By comparison, the promoting effect of increasing pH could be neglected for M3. This was reflected in Figure 6a, where the flux of M3 showed nearly no change with pH values. Furthermore, as pH increased from 10 to 12, M0 exhibited a substantial total flux reduction of 41.82%. In comparison, the composite membranes showed much smaller flux reductions of 19.14% (MF), 6.33% (M1), 3.41% (M2), and 15.63% (M3). This suggested that the modified membranes (MF, M1, M2, and M3) possessed robust, fluorinated surfaces that mitigated hydroxylation. In particular, the incorporation of SiO_2_ nanoparticles on M1, M2, and M3 further reinforced the surface structure, which prevented the membrane surface from hydroxylating under high pH (≥12) conditions. The low total flux of the M3 membrane was attributed to pore blockage caused by excessive SiO_2_ nanoparticles, which restricted water vapor and ammonia transport. Comparative analysis indicated that the M2 membrane exhibited superior alkali resistance, making it the most promising candidate for ammonia–nitrogen recovery from wastewater.

As illustrated in Figure 6b, when the initial ammonia–nitrogen concentration in feed increased from 550 mg/L to 1000 mg/L, the ammonia absorption rates reached 47.46%, 61.77%, 61.77%, 66.32%, and 40.1% for the M0, MF, M1, M2, and M3 membranes, respectively. A decrease in the absorption rate was observed for the M0 membrane. This was because a higher feed concentration required more NaOH to reach the same initial pH value. This increased concentration of NaOH damaged the hydrophobic groups on the pristine membrane surface, exposing numerous hydroxyl groups. In contrast, the performance of the modified membranes was not adversely affected by the increased ammonia nitrogen concentration. Moreover, M2 exhibited a 2.95% increase in absorption rate under these conditions. At the ammonia–nitrogen concentration of 1000 mg/L, the total fluxes of the M1, M2, and M3 membranes remained nearly unchanged. The M3 membrane exhibited a lower overall flux than M0 due to pore blockage, whereas M1 and M2 remained highly stable. These results indicated that increasing ammonia nitrogen concentration had minimal impact on the absorption rate and flux of M1, M2, and M3. In particular, M2 exhibited the highest performance. It could tolerate higher ammonia concentrations and demonstrated a superior resistance to hydroxylation caused by alkali, making it highly suitable for treating high ammonia concentrated wastewater.

As shown in Figure 6c, when the absorbent concentration on the permeate side increased from 0.204 mol/L to 0.51 mol/L, the corresponding ammonia absorption rates for M0, MF, M1, M2, and M3 were 60.33%, 66.52%, 62.23%, 67.68%, and 39.59%, respectively. Compared to the results with the 0.204 mol/L H_2_SO_4_ solution, the absorption rates for all membranes either remained stable or slightly increased. This minor improvement was attributed to the higher acid concentration, which accelerated ammonia absorption and facilitated its transmembrane transfer. Furthermore, increasing the absorbent concentration had no significant effect on total flux for either the pristine membrane or the modified composite membranes. This stability was attributed to acid concentration and had no significant effect on ammonia mass transfer resistance due to the rapid reactional speed between ammonia and sulfuric acid. Furthermore, both the pristine membrane and the modified composite membranes had high corrosion resistance to the increasing sulfuric acid concentration.

### 3.3. Treatment of Actual Wastewater

In this study, M0 and M2 membranes were employed to treat two challenging actual wastewaters with high ammonia–nitrogen concentration: source-separated urine and landfill leachate. The objective was to systematically evaluate the performance of the superhydrophobic composite membrane loading the optimized SiO_2_ capacity (M2) for real wastewater treatment. Based on the results of Section 3.2, the moderate operating conditions were selected for comparison between M0 and M2, including an initial feed pH of 11 and the use of a 0.204 mol/L H_2_SO_4_ solution on the permeate side for ammonia absorption. The operating temperature difference and velocity were consistent with Section 3.2. Before the MD operation, the feed solutions were filtered through a 0.45 μm membrane after pH adjustment with NaOH to eliminate interference from chemical precipitates.

#### 3.3.1. Source-Separated Urine Wastewater Treatment

The source-separated urine for experiments was stored for 7 days (allowing for a sufficient release of ammonia) with its composition detailed in Table 2.

**Figure 7:**
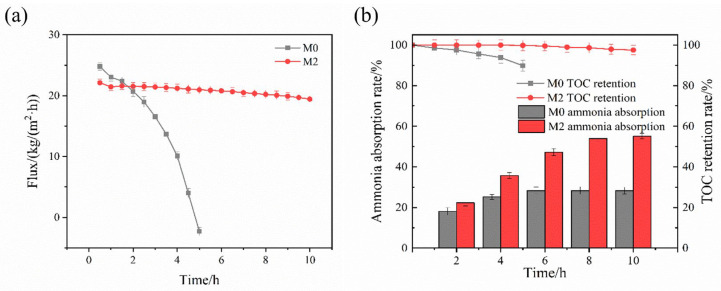
(**a**) Permeate flux and (**b**) ammonia–nitrogen absorption rate and TOC retention rate of source-separated urine treated by the membranes (feed: 500 mL at 70 °C, pH: 11, permeate: 500 mL at 20 °C, H_2_SO_4_: 0.204 mol/L, cross-flow velocity: 0.01 m/s, duration: 10 h).

Figure 7a illustrated the permeate flux of the M0 and M2 membranes during urine distillation. The flux of M0 declined rapidly throughout the experiment, reaching nearly zero after 5 h. In contrast, the M2 membrane maintained a stable flux of 19.43 kg/(m^2^·h) in 10 h, with a total flux loss of only 12.16%. This significant difference in performance was attributed to the high concentration of various organic compounds and salts present in the source-separated urine. These organics adhered to the M0 membrane surface, causing scaling and fouling that blocked pores and eventually led to membrane wetting. However, the M2 membrane’s superhydrophobic properties could effectively resist organic adhering and wetting. As shown in Figure 7b, the performance of the M0 membrane deteriorated significantly over time. After 5 h, as the M0 was wetted, its TOC retention rate dropped to 89.8%, and the mass transfer process was terminated. Its ammonia absorption rate was only 28.3%, which was much lower than that obtained with simulated wastewater (Figure 6a). In contrast, the M2 membrane exhibited excellent stability. After 10 h of operation, M2 achieved a TOC retention rate of 97.5% and an ammonia absorption rate of 55.1%. This absorption rate was nearly twice that of the M0 membrane and only 9.32% lower than its value for simulated wastewater. These results demonstrated that the M2 membrane was highly suitable for treating complex wastewater streams such as source-separated urine.

#### 3.3.2. Landfill Leachate Wastewater Treatment

The composition details of landfill leachate for experiments were provided in Table 3.

**Figure 8:**
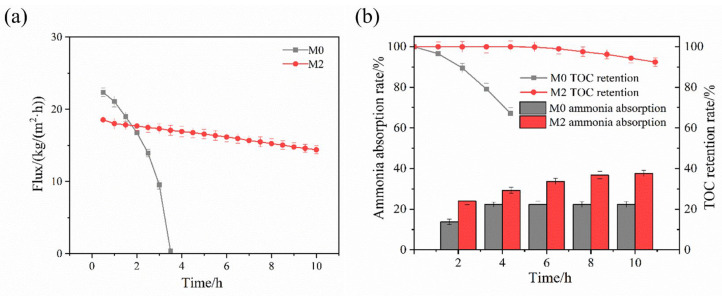
(**a**) Permeate flux and (**b**) ammonia–nitrogen absorption rate and TOC retention rate of landfill leachate treated by the membranes (feed: 500 mL at 70 °C, pH: 11, permeate: 500 mL at 20 °C, H_2_SO_4_: 0.204 mol/L, cross-flow velocity: 0.01 m/s, duration: 10 h).

Figure 8a showed the flux performance of M0 and M2 during DCMD treatment of landfill leachate. The initial flux of the M0 membrane was significantly lower than that observed for the simulated wastewater. Although the M0 membrane began with a slightly higher flux than the M2 membrane, its flux declined rapidly within 3.5 h until mass transfer ceased due to complete membrane wetting. Consequently, its total permeated volume was much lower than that of the M2 membrane. In contrast, the M2 membrane maintained a stable flux throughout the experiment, reaching 14.4 kg/(m^2^·h) after 10 h with a total decline of only 22.4%. The rapid invalidation of the M0 membrane was attributed to the high concentrations of organic contaminants and salts in the leachate, which caused severe fouling on the pristine commercial PVDF surface and pores, leading to rapid wetting. The separation performance results in Figure 8b further demonstrated this difference. At the time point of 3.5 h (invalidation of membrane process), the M0 membrane showed a TOC retention rate of only 67.2% and an ammonia absorption rate of 22.31%. In contrast, after 10 h of continuous operation, the M2 membrane maintained a TOC retention rate of 92.4% and an ammonia absorption rate of 37.58%, about 1.7 times that of the M0.

The composition of contaminants in landfill leachate was more complex, especially with a higher amount of suspended solids, leading to reduced permeate flux. Based on the MD performance for the two actual wastewater, it could be inferred that the superhydrophobic surface of M2 significantly enhanced the membrane’s anti-wetting and anti-fouling capacity. This was mainly due to the optimized SiO_2_ nanoparticle capacity and fluorination modification. In addition, the excellent performance of M2 might be partially attributed to the moderate reduction in membrane pore size caused by modification. Although the initial flux was slightly lower, the smaller pore size of M2 improved the rejection performance and reduced the possibility of contaminant penetration into the membrane pores.

### 3.4. Fundamental Analysis for Anti-Wetting Mechanism of Modified Membranes

**Figure 9:**
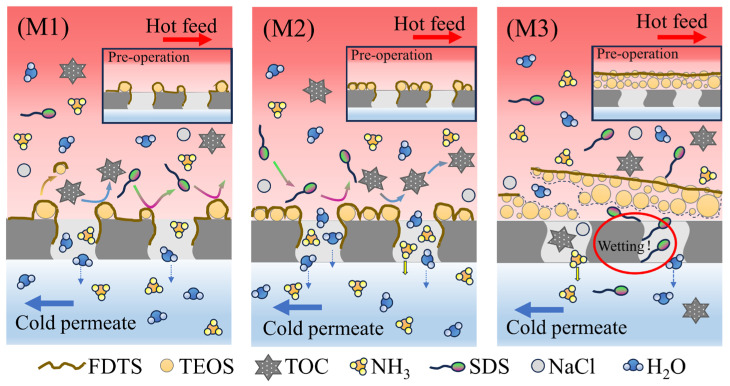
Schematic illustration of the anti-wetting mechanism of the modified membranes.

According to the above results of MD performances for ammonia absorption. M2 displayed the highest wetting resistance. Taking into account the characterization results and anti-wetting performance, the different anti-wetting ability for M1, M2, and M3 could be attributed to the different surface characteristics caused by different surface silicon contents. The schematic representations of the anti-wetting mechanism of M1, M2 and M3 were shown in Figure 9.

The M1 membrane, with its relatively low silica loading, exhibited a sparse distribution of particles on the PVDF surface. This configuration slightly increased surface roughness and moderately improved the water contact angle. Although some coating detachment occurred during operation, M1 still demonstrated a measurable resistance to wetting induced by SDS and organic matter. In contrast, M3 possessed a high silica loading that almost completely covered the membrane surface. Even if this led to a slight increase in water contact angle, the extensive coverage severely reduced flux, yielding the lowest performance among the three. Moreover, as the experiment progressed, the gradual peeling of both silica nanoparticles and the fluorinated layer compromised its antifouling properties, making SDS and organic foulants more easily to contact the pristine PVDF surface. M2, however, presented an optimal balance. Its silica nanoparticles were uniformly distributed, forming a dense and homogeneous coating that conferred stable superhydrophobicity. Consequently, M2 maintained relatively high flux and exhibited excellent, sustained resistance to both SDS permeation in NaCl solution and organic foulant adhesion in real wastewater throughout the experiment. In summary, the M2 membrane, combining moderate loading of SiO_2_, uniform roughness distribution, and robust stability, delivered the best overall performance in terms of water flux, ammonia absorption, and comprehensive anti-wetting capability for the MD process.

## 4. Conclusions

This study aimed to address the critical issues of pore wetting encountered during MD treatment for highly concentrated ammonia wastewater. A superhydrophobic layer was constructed on the surface of a commercial PVDF membrane by depositing SiO_2_ nanoparticles and surface fluorination. The main conclusions were summarized as follows:

(1) Superhydrophobic PVDF membranes (M2) were successfully fabricated through SiO_2_ nanoparticle deposition (6% silicon content) and surface fluorination. M2 exhibited the best performance in contact angle and anti-wetting tests.
(2) All modified membranes exhibited excellent tolerance to high pH conditions during MD ammonia absorption experiments, resulting in enhanced ammonia absorption efficiency.
(3) In MD experiments for real wastewater treatments (source-separated urine and landfill leachate), the M2 membrane exhibited significantly superior stability and anti-wetting properties compared to the pristine PVDF membrane.

## Figures and Tables

**Table 1:** Serial number of different membranes.

Membrane Number	Membrane Type	Modified Methods on Membrane Surface
M0	pristine membrane	--
MF	PVDF/F composite membrane	surface hydroxylated, grafted with fluoride
M1	PVDF/SiO_2_/F composite membrane	surface hydroxylated, content of 1% tetraethyl orthosilicate coated, followed by fluoride grafting.
M2	PVDF/SiO_2_/F composite membrane	surface hydroxylated, content of 6% tetraethyl orthosilicate coated, followed by fluoride grafting.
M3	PVDF/SiO_2_/F composite membrane	surface hydroxylated, content of 12% tetraethyl orthosilicate coated, followed by fluoride grafting.

**Table 2:** Composition of experimental urine components.

Indicators	Numerical Values
Mg^2+^	51 ± 0.5 (mg/L)
NH_3_–N	940 ± 100 (mg/L)
Ca^2+^	84 ± 0.5 (mg/L)
Na^+^	2730 ± 50 (mg/L)
K^+^	2170 ± 200 (mg/L)
TP	430 ± 100 (mg/L)
TOC	4462 ± 342 (mg/L)
pH	6.81 ± 0.5

**Table 3:** Composition of experimental landfill leachate.

Indicators	Numerical Values
TOC	5742 ± 480 (mg/L)
NH_3_–N	573 ± 100 (mg/L)
pH	8.8 ± 0.5
Conductivity	14.7 ± 0.3 (ms/cm)
Soluble phosphate	11.8 ± 0.3 (mg/L)
K^+^	2776 ± 200 (mg/L)
Na^+^	531 ± 30 (mg/L)
Zn	0.7 ± 0.05 (mg/L)
Mg^2+^	33.7 ± 0.5 (mg/L)
Cr	0.8 ± 0.05 (mg/L)
Cu	0.2 ± 0.05 (mg/L)
Mn	1.1 ± 0.05 (mg/L)

## Data Availability

The original contributions presented in this study are included in the article. Further inquiries can be directed to the corresponding authors.

## Supplementary Materials

The following supporting information can be downloaded at: https://www.mdpi.com/article/10.3390/membranes16030100/s1.

## Abbreviations

The following abbreviations are used in this manuscript:

MD	Membrane distillation
DCMD	Direct contact membrane distillation
TEOS	Tetraethyl orthosilicate
SDS	Sodium dodecyl sulfate
TOC	Total organic carbon
FDTS	1H,1H,2H,2H-perfluorodecyltriethoxysilane
TN	Total nitrogen

## References

[1] Alemayehu Y.A., Asfaw S.L., Terfie T.A. (2020). Nutrient recovery options from human urine: A choice for large scale application. Sustain. Prod. Consum..

[2] Damtie M.M., Volpin F., Yao M., Tijing L.D., Hailemariam R.H., Bao T., Park K.-D., Shon H.K., Choi J.-S. (2021). Ammonia recovery from human urine as liquid fertilizers in hollow fiber membrane contactor: Effects of permeate chemistry. Environ. Eng. Res..

[3] Luther A.K., Desloover J., Fennell D.E., Rabaey K. (2015). Electrochemically driven extraction and recovery of ammonia from human urine. Water Res..

[4] Xu L., Chen Y., Wang Z., Zhang Y., He Y., Zhang A., Chen H., Xue G. (2023). Discovering dominant ammonia assimilation: Implication for high-strength nitrogen removal in full scale biological treatment of landfill leachate. Chemosphere.

[5] Amaral M.C.S., Magalhães N.C., Moravia W.G., Ferreira C.D. (2016). Ammonia recovery from landfill leachate using hydrophobic membrane contactors. Water Sci. Technol..

[6] Miao L., Yang G., Tao T., Peng Y. (2019). Recent advances in nitrogen removal from landfill leachate using biological treatments—A review. J. Environ. Manag..

[7] Abdel-Shafy H.I., Ibrahim A.M., Al-Sulaiman A.M., Okasha R.A. (2024). Landfill leachate: Sources, nature, organic composition, and treatment: An environmental overview. Ain Shams Eng. J..

[8] Farsani M.H., Yengejeh R.J., Mirzahosseini A.H., Monavari M., Hassani A.h., Mengelizadeh N. (2021). Effective leachate treatment by a pilot-scale submerged electro-membrane bioreactor. Environ. Sci. Pollut. Res..

[9] Sohn W., Merenda A., Shafaghat A.H., El Saliby I., Zhang Y., Jia X., Guan J., Phuntsho S., Shon H.K. (2025). Influence of temperature and dissolved oxygen on nitrification in a membrane bioreactor treating urine. J. Water Process Eng..

[10] Li Y., Tang F., Xu D., Xie B. (2021). Advances in Biological Nitrogen Removal of Landfill Leachate. Sustainability.

[11] Yan Z., Li A., Shim H., Wang D., Cheng S., Wang Y., Li M. (2022). Effect of ozone pretreatment on biogranulation with partial nitritation—Anammox two stages for nitrogen removal from mature landfill leachate. J. Environ. Manag..

[12] Jalili Jalalieh B., Salehi Pourbavarsad M., Cumbie B., Jackson W.A. (2024). Improving carbon and nitrogen removal efficiency in high-strength nitrogen wastewater via two-stage nitritation-anammox process. J. Environ. Chem. Eng..

[13] Kuleyin A., Yeni O., Sisman Y. (2020). Ammonia and COD Removal from Landfill Leachate Using MAP Precipitation Method. Glob. NEST J..

[14] Tonetti A.L., De Camargo C.C., Guimarães J.R. (2016). Ammonia removal from landfill leachate by struvite formation: An alarming concentration of phosphorus in the treated effluent. Water Sci. Technol..

[15] Kuleyin A., Yeni Ö., Şişman Y. (2020). Removal of ammonia and COD from leachate by MAP precipitation method and contribution of natural materials. Desalination Water Treat..

[16] Dos Santos H.A.P., De Castilhos Júnior A.B., Nadaleti W.C., Lourenço V.A. (2020). Ammonia recovery from air stripping process applied to landfill leachate treatment. Environ. Sci. Pollut. Res..

[17] Reis B.G., Silveira A.L., Lebron Y.A.R., Moreira V.R., Teixeira L.P.T., Okuma A.A., Amaral M.C.S., Lange L.C. (2020). Comprehensive investigation of landfill leachate treatment by integrated Fenton/microfiltration and aerobic membrane bioreactor with nanofiltration. Process Saf. Environ. Prot..

[18] Wu Y. (2018). A Preliminary Study on Application of MBR + NF/RO (Membrane Bio-Reactor + Nanofiltration/Reverse Osmosis) Combination Process for Landfill Leachate Treatment in China. IOP Conf. Ser. Earth Environ. Sci..

[19] Ray H., Perreault F., Boyer T.H. (2020). Rejection of nitrogen species in real fresh and hydrolyzed human urine by reverse osmosis and nanofiltration. J. Environ. Chem. Eng..

[20] Collado S., Núñez D., Oulego P., Riera F.A., Díaz M. (2020). Effect of landfill leachate ageing on ultrafiltration performance and membrane fouling behaviour. J. Water Process Eng..

[21] Kamranvand F., Davey C.J., Williams L., Parker A., Jiang Y., Tyrrel S., McAdam E.J. (2020). Ultrafiltration pretreatment enhances membrane distillation flux, resilience and permeate quality during water recovery from concentrated blackwater (urine/faeces). Sep. Purif. Technol..

[22] Tałałaj I.A. (2022). Performance of integrated sequencing batch reactor (SBR) and reverse osmosis (RO) process for leachate treatment: Effect of pH. J. Environ. Health Sci. Eng..

[23] Gripa E., Campos J.C., da Fonseca F.V. (2021). Combination of ozonation and microfiltration to condition landfill leachate for reverse osmosis treatment. J. Water Process Eng..

[24] Taialaj I.A. (2020). Performance and efficiency of reverse osmosis process in treatment of young and matured landfill leachate. Desalination Water Treat..

[25] García-Pacheco R., Galizia A., Toribio S., Gabarró J., Molina S., Landaburu-Aguirre J., Molina F., Blandin G., Monclús H., Rodríguez-Roda I. (2022). Landfill Leachate Treatment by Using Second-Hand Reverse Osmosis Membranes: Long-Term Case Study in a Full-Scale Operating Facility. Membranes.

[26] Ray H., Perreault F., Boyer T.H. (2022). Ammonia recovery and fouling mitigation of hydrolyzed human urine treated by nanofiltration and reverse osmosis. Environ. Sci. Water Res. Technol..

[27] Wang Y., Li T., Zhu J. (2022). Study on treatment of wastewater with low concentration of ammonia-nitrogen by vacuum plate membrane distillation technology. Water Sci. Technol..

[28] Shirazi M.M.A., Dumée L.F. (2022). Membrane distillation for sustainable wastewater treatment. J. Water Process Eng..

[29] Sim L.N., Jayaraman P., Lau Y.H., Chong T.H., Wang R. (2024). Pharmaceutical wastewater treatment using direct contact membrane distillation. J. Water Process Eng..

[30] Reddy A.S., Kalla S., Murthy Z.V.P. (2022). Nano-particles enhanced hydrophobic membranes: High-performance study for dye wastewater treatment using membrane distillation. J. Water Process Eng..

[31] Yang R., Zhao R., Liu Y., Wu Q., Zhong Z., Liu H., Liu C., Zuo K. (2025). A novel composite membrane for zero liquid discharge of coking wastewater in membrane distillation. Chem. Eng. J..

[32] Hamdan S., Al-Ghafri B., Al-Obaidani S., Tarboush B.A., Al-Sabahi J., Al-Abri M. (2024). Dairy wastewater treatment with direct-contact membrane distillation in Oman. Desalination Water Treat..

[33] Yadav A., Yadav P., Labhasetwar P.K., Shahi V.K. (2021). CNT functionalized ZIF-8 impregnated poly(vinylidene fluoride-co-hexafluoropropylene) mixed matrix membranes for antibiotics removal from pharmaceutical industry wastewater by vacuum membrane distillation. J. Environ. Chem. Eng..

[34] Zhong W., Guo L., Ji C., Dong G., Li S. (2021). Membrane distillation for zero liquid discharge during treatment of wastewater from the industry of traditional Chinese medicine: A review. Environ. Chem. Lett..

[35] Fang D., Amiruddin D.M., Kao I., Mahajan D., Chen X., Hsiao B.S. (2024). Towards the Optimization of a Photovoltaic/Membrane Distillation System for the Production of Pure Water. Membranes.

[36] Amiruddin D., Mahajan D., Fang D., Wang W., Wang P., Hsiao B.S. (2023). A Facile Ultrapure Water Production Method for Electrolysis via Multilayered Photovoltaic/Membrane Distillation. Energies.

[37] Mao Y., Xu J., Chen H., Liu G., Liu Z., Cheng L., Guo Y., Liu G., Jin W. (2023). Hydrophobic metal-organic framework@graphene oxide membrane with enhanced water transport for desalination. J. Membr. Sci..

[38] Lee W.J., Ng Z.C., Hubadillah S.K., Goh P.S., Lau W.J., Othman M.H.D., Ismail A.F., Hilal N. (2020). Fouling mitigation in forward osmosis and membrane distillation for desalination. Desalination.

[39] Jawed A.S., Nassar L., Hegab H.M., van der Merwe R., Al Marzooqi F., Banat F., Hasan S.W. (2024). Recent developments in solar-powered membrane distillation for sustainable desalination. Heliyon.

[40] Alsebaeai M.K., Ahmad A.L. (2020). Membrane distillation: Progress in the improvement of dedicated membranes for enhanced hydrophobicity and desalination performance. J. Ind. Eng. Chem..

[41] Ali E., Orfi J., AlAnsary H., Soukane S., Elcik H., Alpatova A., Ghaffour N. (2021). Cost analysis of multiple effect evaporation and membrane distillation hybrid desalination system. Desalination.

[42] Liu C., Zhu L., Ji R. (2022). Direct contact membrane distillation (DCMD) process for simulated brackish water treatment: An especial emphasis on impacts of antiscalants. J. Membr. Sci..

[43] Simoni G., Kirkebæk B.S., Quist-Jensen C.A., Christensen M.L., Ali A. (2021). A comparison of vacuum and direct contact membrane distillation for phosphorus and ammonia recovery from wastewater. J. Water Process Eng..

[44] Drioli E., Ali A., Macedonio F. (2015). Membrane distillation: Recent developments and perspectives. Desalination.

[45] Xiao Z., Guo H., He H., Liu Y., Li X., Zhang Y., Yin H., Volkov A.V., He T. (2020). Unprecedented scaling/fouling resistance of omniphobic polyvinylidene fluoride membrane with silica nanoparticle coated micropillars in direct contact membrane distillation. J. Membr. Sci..

[46] Lu X., Peng Y., Ge L., Lin R., Zhu Z., Liu S. (2016). Amphiphobic PVDF composite membranes for anti-fouling direct contact membrane distillation. J. Membr. Sci..

[47] Kumar R., Ahmed M., Bhadrachari G., Al-Mesri A., Thomas J.P. (2019). Hydrophobically modified silica blend PVDF nanocomposite membranes for seawater desalination via direct contact membrane distillation. Desalination Water Treat..

[48] Nambikkattu J., Jacob Kaleekkal N. (2023). Investigating the performance of surface-engineered membranes for direct contact membrane distillation. Sep. Sci. Technol..

[49] Jin Y., Wei Z., Meng X., Li J. (2023). Fabrication of superhydrophobic PVDF membrane via catechol/polyamine co-deposition and SiO_2_ nanoparticles for membrane distillation. J. Environ. Chem. Eng..

[50] Xu Y., Qiu Z., Chen J., Ma B., Zou W., Yang Z., Dai R. (2025). Orderly stacked 3D-nanohelices interlayer boosts performance of reverse osmosis membranes for effective water purification. Energy Environ. Sustain..

[51] Qing W., Wu Y., Li X., Shi X., Shao S., Mei Y., Zhang W., Tang C.Y. (2020). Omniphobic PVDF nanofibrous membrane for superior anti-wetting performance in direct contact membrane distillation. J. Membr. Sci..

[52] Zhang P., Xiang S., Gonzales R.R., Li Z., Chiao Y.-H., Guan K., Hu M., Xu P., Mai Z., Rajabzadeh S. (2024). Wetting-and scaling-resistant superhydrophobic hollow fiber membrane with hierarchical surface structure for membrane distillation. J. Membr. Sci..

[53] Xu B., Yang X., Zhou W., Chen F., Zhang X., Zhang X., Zhu X. (2025). Superhydrophobic nanostructured wood membrane for thermal distillation desalination. J. Membr. Sci..

[54] Cha H., Gu B., Jeong S. (2024). Enhancing ammonia selectivity in membrane distillation: The role of membrane structural characteristics. Desalination.

[55] Hu J., Harandi H.B., Liu S., Zhang Y., He T. (2024). Scaling and wetting resistant silica nanoparticle grafted multi-scale corrugated omniphobic membranes for membrane distillation. Desalination.

[56] Zheng G., Yao L., You X., Liao Y., Wang R., Huang J.J. (2021). Effects of different secondary nano-scaled roughness on the properties of omniphobic membranes for brine treatment using membrane distillation. J. Membr. Sci..

[57] Lu K.-J., Zuo J., Chung T.-S. (2016). Tri-bore PVDF hollow fibers with a super-hydrophobic coating for membrane distillation. J. Membr. Sci..

[58] Li X., Shan H., Cao M., Li B. (2019). Facile fabrication of omniphobic PVDF composite membrane via a waterborne coating for anti-wetting and anti-fouling membrane distillation. J. Membr. Sci..

[59] Xiong Z., Lai Q., Lu J., Qu F., Yu H., Chen X., Zhang G., Zhang W., Zhao S. (2023). Silanization enabled superhydrophobic PTFE membrane with antiwetting and antifouling properties for robust membrane distillation. J. Membr. Sci..

[60] Fan H., Gao A., Zhang G., Zhao S., Cui J., Yan Y. (2020). A facile strategy towards developing amphiphobic polysul fone membrane with double Re-entrant structure for membrane distillation. J. Membr. Sci..

[61] Samadi A., Ni T., Fontananova E., Tang G., Shon H., Zhao S. (2023). Engineering antiwetting hydrophobic surfaces for membrane distillation: A review. Desalination.

[62] Capizzano S., Frappa M., Macedonio F., Drioli E. (2021). A review on membrane distillation in process engineering: Design and exergy equations, materials and wetting problems. Front. Chem. Sci. Eng..

