# Supplementary material for: Anti-Wetting PVDF Membrane Modification by Coating Fluoride and Deposing Different Silicon Contents for Membrane Distillation Treatment of Ammonia Nitrogen Wastewater
Source: Membranes (Basel). 2026 Mar 6;16(3):100. doi: 10.3390/membranes16030100 (PMC13027501; doi:10.3390/membranes16030100)
Supplement: Supplementary file 1 [file membranes-16-00100-s001.zip › membranes-4082075-supplementary.pdf]

Supporting Information

Anti-wetting PVDF membrane modification by coating fluoride and deposing different silicon contents for membrane distillation treatment of ammonia nitrogen wastewater

Membranes

Qianliang Liu[1], Xin Guo[1], Hengyu Ai[1,*], Hongbo Liang[2], Fen Li[1], Caihong Liu[3,*]

[1] Key Laboratory of Green Chemical Engineering and Technology of Heilongjiang Province, School of Material Science and Chemical Engineering, Harbin University of Science and Technology, Harbin 150080, China; liuqianliang1982@126.com (Q.L.); 15945362821@163.com (X.G.); lifen@hrbust.edu.cn (F.L.)

[2] School of Mining Engineering, Heilongjiang University of Science and Technology, Harbin 150022, China; lhbhljkjdx@163.com

[3] Key Laboratory of Eco-Environments in Three Gorges Reservoir Region, Ministry of Education, College of Environment and Ecology, Chongqing University, Chongqing 400044, China

* Correspondence: aihengyu@hrbust.edu.cn (H.A.); caihong.liu@cqu.edu.cn (C.L.)

Texts

Text S1.

The preparation of silica gel and fluoride solution.

The silica gel was prepared as follows: 1 mL, 6 mL, and 12 mL of tetraethyl orthosilicate (TEOS, ≥98.0%) were dissolved in mixed solution of anhydrous ethanol and ammonia water and the total volume was adjusted to 100 mL. these mixtures were placed in a 50°C shaking water bath and stirred for 2 hours. The volume ratios of TEOS in the 100 mL mixtures were 1%, 6%, and 12%, respectively. Stop shaking after the additions are complete and continue the reaction in a 60 °C water bath for 24 hours until the SiO_2 gel forms.

The fluoride solution was prepared by adding 5 mL deionized water, 0.5 mL 1H,1H,2H,2H-perfluorodecyltriethoxysilane (FDTS, 96%) and 44.5 ml anhydrous ethanol to a beaker, followed by magnetic stirring at 25 °C for 30 min. The mixture was then transferred to a 60 °C water bath and heated for 18 h to yield the fluoride solution.

Text S2

Calculation Formulas for R_a and R_q

R_a:

$$R_a=\frac{1}{n}\sum|Z_i| \quad (1)$$

Where R_a is the average roughness, n is the number of sampling points, and Z_i is the height of the i-th sampling point.

R_q:

$$R_q=\sqrt{\left(\frac{1}{n}\sum(Z_i\text{-}Z)^2\right)} \quad (2)$$

Where R_q is the root means square roughness, n is the number of sampling points, and Z is the mean height of all sampling points.

Text S3.

The DCMD experimental details.

The membrane module served to separate water and ammonia–nitrogen. The circulation system maintained the transmembrane temperature gradient driving the distillation process, with the feed and permeate streams circulated by a peristaltic pumps. Feed water was heated in one thermostatic bath, while permeate water was cooled in the other one. Thermocouple thermometers were positioned at the inlet and outlet of the membrane module for monitoring solution temperatures. The weight monitoring system recorded water evaporation, while a conductivity meter continuously recorded permeate conductivity. Ammonia nitrogen concentration was measured using a spectrophotometer.
